# *Quantum Materials*: A New Open Section in Materials

**DOI:** 10.3390/ma14123142

**Published:** 2021-06-08

**Authors:** Heesun Yang

**Affiliations:** Department of Materials Science and Engineering, Hongik University, Seoul 04066, Korea; hyang@hongik.ac.kr

*Quantum Materials* is a new open section of *Materials* aimed at publishing original and review articles on novel scientific and applied research that significantly contribute to the understanding and discovery of quantum materials and related phenomena, functions, and applications. Quantum materials can be classically defined as solids with exotic physical properties stemming from the quantum mechanical properties of their constituent electrons [1]. Although quantum materials can be classified as an emerging field, their representative properties have been long known, such as superconductivity and ferromagnetism. Non-trivial properties of quantum materials are revealed to be no longer restricted to strongly correlated electrons in solids that can produce novel functions due to the emergence of their collective behaviors. As new properties and phenomena of quantum materials keep emerging, enabled by contemporary advanced science and technology, their realm is becoming broader.

Quantum materials are at the forefront of contemporary physics and materials science, as they offer a valuable platform to explore the complex interplay among various factors of electron–electron correlation, spin–orbit interaction, symmetry, topology of wavefunctions, low dimensionality, quantum confinement, quantum coherence, quantum fluctuation, and so forth, thus holding promise for next-generation electronic/photonic and energy technologies with currently unavailable functionalities [1,2]. Discovering and manipulating the materials possessing unprecedented quantum properties are of great importance alongside their theoretical understanding. Typical classes of quantum materials include transition metal oxides, Fe-/Cu-based high-temperature superconductors, van der Waals semiconductors, topological insulators, and Weyl semimetals and graphene [2].

Research interest to Section *Quantum Materials* includes, but is not limited to, the following: **superconducting materials**: novel superconductors, cuprates, iron-based systems, and heavy fermion superconductors; **correlated electronic materials**: Mott insulators, magnetism in correlated electron systems, colossal magnetoresistance, multiferroicity and multiferroics, and related theories and methods; **topological quantum materials**: topological insulators/superconductors, Dirac semimetals and Weyl semimetals, magnetic topological insulators, and topological heterostructures and devices; **quantum phenomena in advanced materials:** finite-size and low-dimensional (quantum dot, quantum well, quantum wire, superlattice) systems, photovoltaic/light-emitting systems, photocatalysis, and advanced energy generation systems.

## References

[1] Samarth N. (2017). Quantum materials discovery from a synthesis perspective. Nat. Mater..

[2] Basov D.N., Averitt R.D., Hsieh D. (2017). Towards properties on demand in quantum materials. Nat. Mater..

